# Lack of association between the *VEGFA* gene polymorphisms and preterm birth in Korean women

**DOI:** 10.5808/gi.22064

**Published:** 2023-09-27

**Authors:** Yue Shi, Hyung Jun Kim, Seong Yong Kim, Ga Eun Kim, Han Jun Jin

**Affiliations:** Department of Biological Sciences, College of Science & Technology, Dankook University, Cheonan 31116, Korea

**Keywords:** genetic association, Korean women, polymorphisms, preterm birth, *VEGFA*

## Abstract

Preterm birth (PTB), a pregnancy-related disease, is defined as a birth before 37 weeks of gestation. It is a major cause of maternal mortality and morbidity worldwide, and its incidence rate is steadily increasing. Various genetic factors can contribute to the etiology of PTB. Vascular endothelial growth factor A (*VEGFA*) gene is an important angiogenic gene and its polymorphisms have been reported to be associated with PTB development. Therefore, we conducted a case-control study to evaluate the association between *VEGFA* rs699947, rs2010963, and rs3025039 polymorphisms and PTB in Korean women. A total of 271 subjects (116 patients with PTB and 155 women at ≥38 weeks of gestation) were analyzed in this study. The genotyping of *VEGFA* gene polymorphisms was performed using polymerase chain reaction–restriction fragment length polymorphism. No significant association between the patients with PTB and the control groups was confirmed. In the combination analysis, we found a significant association between PTB and *VEGFA* rs699947 CC-rs2010963 GG-rs3025039 CC combination (odds ratio, 3.77; 95% confidence interval, 1.091 to 13.032; p = 0.031). The *VEGFA* rs699947, rs2010963, and rs3025039 polymorphisms might have no genetic association with the pathogenesis of PTB in Korean women. However, the combination analysis indicates the possibility that *VEGFA* acts in PTB pathophysiology. Therefore, larger sample sets and replication studies are required to further elucidate our findings.

## Introduction

Preterm birth (PTB) refers to pregnancy-related disorders that are delivered before 37 weeks of gestation [1,2]. PTB is largely responsible for the deaths of children [3]. According to a previous study, approximately 15 million babies are born preterm yearly, and the prevalence rate is approximately 5%–18% [4]. In South Korea, PTB accounted for 7.2% of births in 2016, a 1.5-fold increase from 2006 [5]. However, the exact causes of PTB remain unclear [6].

Various studies have shown that PTB is a syndrome caused by multiple mechanisms including inflammatory infection and uteroplacental ischemia (UPI) [7-10]. Particularly, the causes of UPI are mostly due to the abnormal pattern of the placental stem artery development, accompanied by an insufficient increase in capillary angiogenesis, and terminal villous dysplasia [11]. The main pathways involved in vascular dysplasia are vascular endothelial growth factor (VEGF), angiopoietin-TIE2, and transforming growth factor-β [12]. Among them, the VEGF family-mediated angiogenesis plays a key role [13-16]. VEGF is an angiogenic factor that also participates in the formation of blood vessels during the development of the corpus luteum [17]. Additionally, a previous study reported on the study of *in vivo* model systems showed that VEGF could regulate circulating progesterone levels [18]. Moreover, blocking of VEGF second-trimester signal causes impairment of luteal circulation and induces PTB [19,20]. Also, the American College of Obstetricians and Gynecologists suggested that the progesterone supplement is a useful way to reduce the risk of PTB [21].

In the VEGF family, vascular endothelial growth factor A (VEGFA) is an angiogenic factor for developing the embryonic vasculature [22]. Related studies of maternal serum levels found that an increase in *VEGFA* would promote PTB development [23,24]. Additionally, the *VEGFA* polymorphisms have been reported to be associated with altered *VEGFA* protein expression [25,26]. *VEGFA* is located on chromosome 6 (6p12) [27]. Papazoglou et al. [28] confirmed the association between rs2010963 in *VEGFA* and spontaneous preterm births (sPTB). Also, a previous study has shown that *VEGFA* rs699947 polymorphism is associated with the risk of sPTB [20]. A genetic-environmental study also confirmed that rs3025039 is associated with the risk of PTB [29]. However, to the best of our knowledge, a genetic association study between *VEGFA* and PTB has not been conducted on Korean women. Therefore, we investigated the genetic correlation between the three *VEGFA* gene polymorphisms (rs699947, rs2010963, and rs3025039) and PTB in Korean women. We also analyzed the correlation of three polymorphisms of *VEGFA* with characteristic data.

## Methods

### Subject

We analyzed a total of 271 samples that include 116 patients with PTB and 155 control groups recruited by the Department of Obstetrics and Gynecology of Dankook University Hospital in Korea (Table 1). In the screening of experimental subjects, PTB patients were composed of women who gave birth before 37 weeks, and the control groups were composed of women who gave birth after 37 weeks. The patients with PTB and the control groups did not include women with multiple pregnancy, gestational diabetes mellitus, pre-gestational hypertension, and early placental separation. The study was conducted after approval by the Institutional Review Board of Dankook University Hospital F (date of approval and the project identification code are respectively 16 November 2017 and DKUH 2016-12-003-005).

### DNA extraction and genotyping

**Fig. 1.**
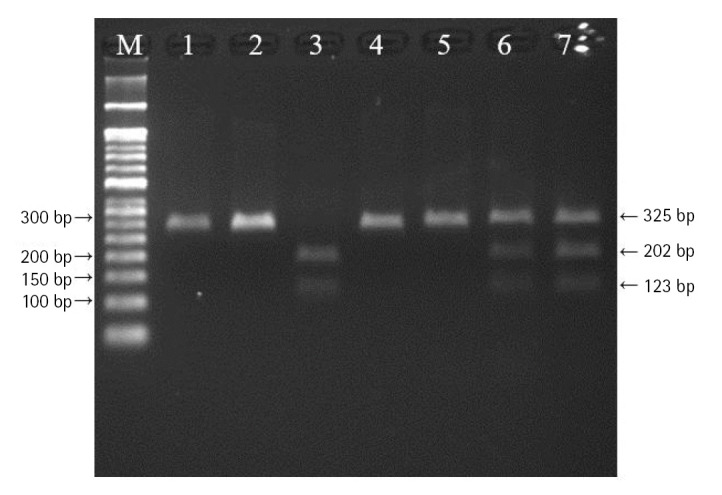
The polymerase chain reaction–restriction fragment length polymorphism result of vascular endothelial growth factor A (*VEGFA*) rs699947 polymorphism. Lanes are as follows: M, 50 bp DNA size ladder; 1, 2, 4, 5, CC genotype; 3, AA genotype; 6, 7, CA genotype.

**Fig. 2.**
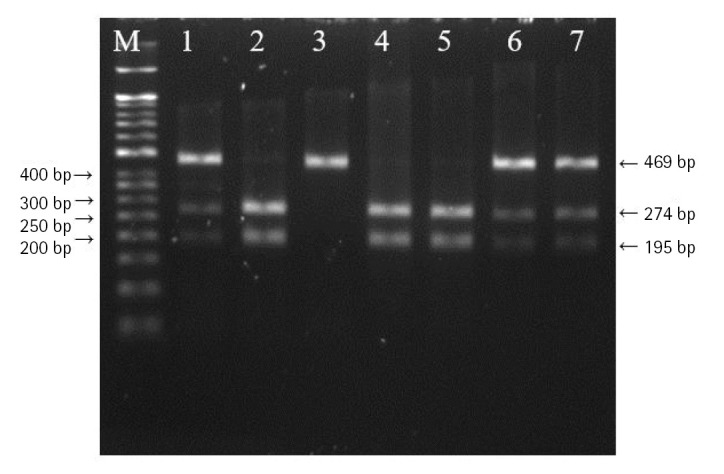
The polymerase chain reaction–restriction fragment length polymorphism result of vascular endothelial growth factor A (*VEGFA*) rs2010963 polymorphism. Lanes are as follows: M, 50 bp DNA size ladder; 1, CC genotype; 2, 3, 6, 7, GC genotype; 4, 5, GG genotype.

**Fig. 3.**
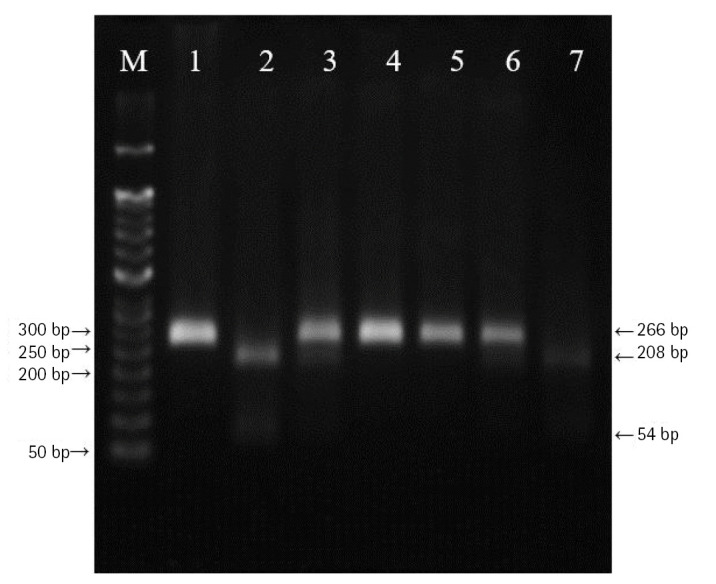
The polymerase chain reaction–restriction fragment length polymorphism result of vascular endothelial growth factor A (*VEGFA*) rs3025039 polymorphism. Lanes are as follows: M, 50 bp DNA size ladder; 1, 4, 5, CC genotype; 3, 6, CT genotype; 2, 7, TT genotype.

DNA was extracted from peripheral blood or buccal cells using the GeneAll Exgene Clinic SV mini kit (GeneAll, Seoul, Korea). Genotypes of *VEGFA* rs699947, rs2010963, and rs3025039 polymorphisms were detected using the polymerase chain reaction–restriction fragment length polymorphism. Primer sets were designed for a determination of rs699947, rs2010963, and rs3025039 polymorphisms reported by previous studies (Table 2) [30-32]. Each polymerase chain reaction (PCR) reaction was performed in a total volume of 20 μL mixture containing 10 ng of genomic DNA, 10 pM of each primer, 0.2 mM MgCl_2_, 10× PCR buffer, and 1.0 U NV DNA polymerase (NAVI BioTech, Cheonan, Korea). The PCR amplifications were conducted with a C1000 Touch thermal cycler (Bio-Rad, Hercules, CA, USA) under the following conditions: 95℃ for 5 min, followed by 32 cycles at 95℃ for 30 s, 61℃ annealing 30 s, and 72℃ for 50 s and then a final extension at 72°C for 10 min for rs699947; 95℃ for 5 min, followed by 35 cycles at 94℃ for 1 min, 62℃ annealing 1 min, and 72℃ for 1 min and then a final extension at 72°C for 5 min for rs2010963; 94℃ for 3 min, followed by 35 cycles at 94℃ for 30 s, 58℃ annealing 30 s, and 72℃ for 30 s and then a final extension at 72°C for 10 min for rs3025039. Each of the PCR product was digested with 1.0 U *Bg*lⅡ (rs699947 C>A), *Bsm*FI (rs2010963 C>G), and *Nla*Ⅲ (rs3025039 C>T) restriction enzymes (Enzynomics, Daejeon, Korea) for 6 h at 37°C (*Bg*lⅡ and *Nla*Ⅲ) and 65°C (*Bsm*FI), respectively. The polymorphic BglII site for rs699947 produced 325 bp (C allele) or 202 bp and 123 bp (A allele) fragments (Fig. 1). The restricted alleles of rs2010963 were 469 bp (C allele) or 274 bp and 195 bp (G allele) fragments, and rs3025039 was confirmed by different fragment sizes of 266 bp (C allele) or 208 bp and 54 bp (T allele) (Figs. 2 and 3).

### Data analysis

A test of cross tabulation analysis was performed using the SPSS 26 Statistics (IBM Corp., Armonk, NY, USA). The chi-squared tests were used to assess the Hardy-Weinberg equilibrium. We used SISA (Simple Interactive Statistical Analysis, https://www.quantitativeskills.com/sisa/) to compare the genotype or allele frequencies between groups through a 2 × r table and to calculate odds ratio (OR) with 95% confidence intervals (CIs) using a 2 × 2 table. Based on genotype data, linkage disequilibrium (LD) was estimated using the HaploView 4.2. Haplotype analysis was performed to compare haplotype frequencies between the patients with preterm and control groups by HAPSTAT software v.3.0. In addition, combination analyses of genotype frequency were performed using the SNPstats web-based tool (SNPstats, https://www.snpstats.net/snpstats/). A p-value of <0.05 was considered statistically significant. Bonferroni correction was applied to adjust for multiple comparisons [33].

## Results

In this study, 271 pregnant women (including 116 patients with PTB and 155 control groups) were analyzed. The mean values of age, height, weight, pre- and post-pregnancy weight, systolic blood pressure, and diastolic blood pressure were insignificantly different between the patients with PTB and control groups (p > 0.05). However, a significant difference was found in birth weight and gestational age between patients with PTB and control groups (p < 0.05) (Table 1).

Genotype and allele distributions of *VEGFA* gene polymorphisms for the 116 patients with PTB and 155 controls are presented in Table 2. The genotype and allele frequencies of rs699947, rs2010963, and rs3025039 were insignificantly associated with PTB (p > 0.05) (Tables 3 and 4).

**Fig. 4.**
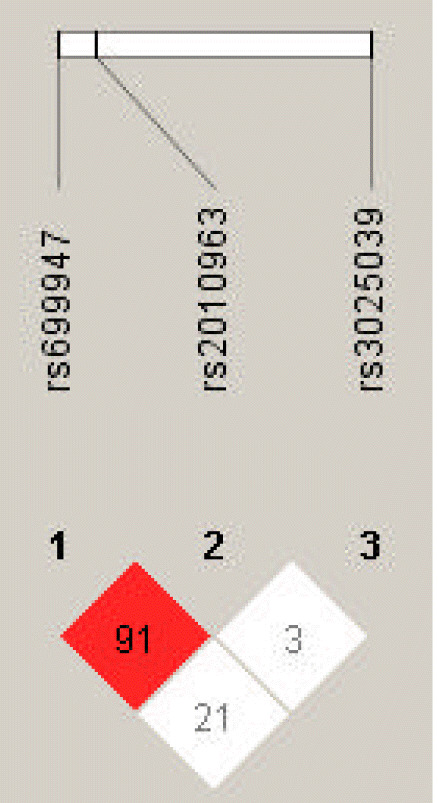
Linkage disequilibrium (LD) plot of single nucleotide polymorphisms of vascular endothelial growth factor A (*VEGFA*). Numbers represent LD between markers based on the D′ values.

LD analysis was performed for rs699947, rs2010963, and rs3025039 polymorphisms in patients with PTB and controls. The LD between rs699947 and rs2010963 was high (D’ = 0.91, r^2^ = 1.0) (Fig. 4). Therefore, we analyzed the frequencies of the four common haplotypes. No significant difference was found between the rs699947 and rs2010963 polymorphism and PTB incidence (p > 0.05) (Supplementary Table 1).

In the genotype combination analysis of the three gene polymorphisms (rs699947 C>A, rs2010963 C>G, and rs3025039 C>T), we found a significant association with PTB from rs699947 CC-rs2010963 GG-rs3025039 CC combination (OR, 3.77; 95% CI, 1.091 to 13.032; p = 0.031) (Table 5).

## Discussion

The VEGF protein levels increase during late pregnancy and peak during the time of cervical ripening [34]. It is reported that higher VEGF protein expression in the early stages could lead to premature cervical ripening, resulting in PTB [34,35]. Several studies reported that *VEGFA* polymorphisms are associated with altered VEGF production [25]. Among them, the rs699947 CC homozygotes and rs2010963 C allele were associated with higher secretion of VEGF [23]. Moreover, in carriers of the rs3025039 T allele, VEGF plasma levels were significantly lower [26]. Since *VEGFA* genetically controls VEGF protein expression [36-38]. We analyzed the genetic associations between *VEGFA* rs699947, rs2010963, and rs3025039 polymorphisms and PTB in Korean women.

The influence of *VEGFA* gene polymorphisms on PTB has been inconsistent to date, possibly because of ethnic heterogeneity [39]. In this analysis, no significant association between rs2010963 polymorphism and PTB was found (p > 0.05) (Tables 3 and 4). Contrarily, *VEGFA* rs2010963 G allele was significantly associated with PTB in the Malaysian population [20]. The minor allele frequency (MAF) of South Asian population is different from that of Korean population (Table 6). However, rs699947 and rs2010963 are also in a strong LD in Malaysians (D′ = 0.97, r^2^ = 1.0) [20]. Therefore, we carefully speculate that the significant association was not replicated because of the genetic heterogeneity. For evaluating the effect of these two polymorphisms in *VEGFA* for PTB, further studies need to be examined in different populations. Also, in the results of *VEGFA* rs699947 and rs3025039, no significant association in the Korean population (p > 0.05) (Table 3 and 4). This is consistent with the findings of Langmia et al. [20], which suggested the *VEGFA* rs699947 and rs3025039 were insignificantly associated with PTB in the Malaysian population. Contrastively, the rs699947 AA genotype was associated with a risk of PTB in Australian and New Zealand populations [29]. Also, rs3025039 CT and TT genotype of *VEGFA* was associated with an increased risk of sPTB in the Greek population [28]. MAFs for each *VEGFA* polymorphism were different in Korea population (in East Asia population), Europe, and South Asia population (Table 6). Also, each population is genetically distinct [40]. It can be seen that the effect of *VEGFA* gene on PTB is not consistent in different populations, which may be due to the genetic heterogeneity [39]. Zhao et al. [29] reported an increased risk of PTB in women with the *VEGFA* rs3025039 CC genotype among the women exposed to higher air quality index levels. Although the study considered the influence of environmental factors, it also confirmed that rs3025039 was potentially associated with PTB pathogenesis [29]. Therefore, more studies in other populations are needed to determine the association of *VEGFA* gene polymorphisms with PTB development.

Haplotype analysis provides more information about identifying key candidate genes for PTB than testing a single locus, and it reduces the false-positive associations in the result [41,42]. Hence, we performed the haplotype analysis of *VEGFA* rs2010963, rs699947, and rs3025039 by LD analysis (Fig. 4). The LD between rs2010963 and rs699947 was high (D′ = 0.91). The rs699947 CC-rs2010963 GG combination showed a marginal trend toward significance (OR, 0.36; 95% CI, 0.125 to 1.021; p = 0.051). However, no significant result in the haplotype analysis was found (Supplementary Table 1). In contrast, in the genotype combination analysis, the rs699947 CC-rs2010963 GG-rs3025039 CC combination was associated significantly with patients with PTB (OR, 3.77; 95% CI, 1.091 to 13.032; p = 0.031) (Table 5). According to this result, we may speculate that the *VEGFA* rs699947 CC-rs2010963 GG-rs3025039 CC combination plays an etiological role in PTB development in Korean women. However, the significant results were not maintained after correction for multiple testing. Therefore, *VEGFA* polymorphisms, which were analyzed in this study, were not associated with PTB in Korean women.

This study has several limitations. Firstly, the sample size used in this study was relatively small. However, in the statistical analysis using the G*Power program, sample power of over 90% was obtained in 271 subjects, exceeding the minimum limit (80%) for clinical studies [43]. Nonetheless, more samples would be requested to validate our results. Secondly, only one gene was investigated in this study, and gene-gene or gene-environment interactions were not considered. PTB is generally not caused by the influence of a single therapeutic gene or an environmental factor [44]. Thus, it was concluded that *VEGFA* and other genes work together to cause PTB. Finally, this study included only Korean women. Since we do not replicate a significant association between polymorphisms in *VEGFA* and PTB, further studies conducted in various populations will be required. Research of the wider populations can reveal missed insights into the association between genetic markers and phenotype [45]. Analysis in other populations is critical to assess the accuracy and wider relevance of a finding. Hence, this study is limited to Korea and may need to be replicated in other countries, including different populations allowing assessment of potential racial or ethnic heterogeneity.

Despite these limitations, this study is the first analysis of the association between *VEGFA* gene polymorphisms and PTB in Korean women. Conclusively, our study could not confirm genetic relationships between the *VEGFA* rs699947, rs2010963, and rs3025039 polymorphisms, and PTB pathogenesis. Therefore, current evidence does not support using the *VEGFA* to predict risk or as a marker for therapeutic intervention in Korea. Replication studies with larger sample sizes and different populations are still needed to elucidate the genetic relationship between the *VEGFA* gene polymorphisms and the pathogenesis of PTB.

## Figures and Tables

**Table 1.** Characteristics of patients with preterm birth and control groups

Characteristic	Preterm birth (n=116)	Control (n=155)	p-value^a^
Age (y)	30.59 ± 4.59	30.41 ± 4.91	0.779
Heigh (cm)	160.29 ± 4.59	160.56 ± 5.56	0.697
Pre-pregnant weight (g)	56.82 ± 9.92	56.1 ± 10.41	0.599
Post-pregnant weight (g)	67.37 ± 10.51	69.46 ± 10.45	0.140
SBP (mmHg)	120.24 ± 14.97	121.32 ± 14.47	0.585
DBP (mmHg)	74.76 ± 12.82	75.79 ± 11.87	0.535
Birth weight (g)	2,354.51 ± 643.04	3,158.46 ± 463.68	< 0.001^*^
GA (wk)	33.19 ± 3.06	38.74 ± 1.06	< 0.001^*^

Values are presented as mean ± standard deviation. SBP, systolic blood pressure; DBP, diastolic blood pressure; GA, gestational age at delivery (week).

* p < 0.05.

a The p-value is for t-test.

**Table 2.** PCR primer sequences, annealing temperature and the expected sizes of PCR products

Maker	Primer sequence	Annealing temperature (°C)	Expected fragments sizes (bp)	
*VEGFA *rs699947 (C>A)	F: 5′-AGGGTTCGGGAACCAGATC-3′	61	325	
	R: 5′-CTCGGTGATTTAGCAGCAAG-3′			
*VEGFA *rs2010963 (C>G)	F: 5′-TTGCTTGCCATTCCCCACTTGA-3′	62	469	
	R: 5′-CCGAAGCGAGAACAGCCCAGAA-3′			
*VEGFA *rs3025039 (C>T)	F: 5′-AGGGTTTCGGGAACCAGATC-3′	58	266	
	R: 5′-CTCGGTGATTTAGCAGCAAG-3′			

PCR, polymerase chain reaction; *VEGFA*, vascular endothelial growth factor A.

**Table 3.** Genotype and allele frequencies distribution of *VEGFA* gene polymorphisms in patients with preterm birth and control group

Maker			Genotype/allele distribution, n (%)		
			Preterm birth (n = 116)	Control (n = 155)	p-value^a^
*VEGFA* rs699947	Genotype	CC	67 (57.8)	78 (50.3)	0.366
		CA	41 (35.3)	68 (43.9)	
		AA	8 (6.9)	9 (7.8)	
	HWE		0.617	0.240	
	Allele	C	175 (75.4)	242 (73.8)	0.407
		A	57 (24.6)	86 (26.2)	
rs2010963	Genotype	CC	24 (20.7)	32 (20.6)	0.155
		CG	51 (44.0)	84 (54.2)	
		GG	41 (35.3)	39 (25.2)	
	HWE		0.275	0.284	
	Allele	C	99 (42.7)	148 (47.7)	0.241
		G	133 (57.3)	162 (52.3)	
rs3025039	Genotype	CC	81 (69.8)	102 (65.8)	0.490
		CT	31 (26.7)	50 (32.3)	
		TT	4 (3.4)	3 (1.9)	
	HWE		0.632	0.264	
	Allele	C	193 (83.2)	254 (81.9)	0.704
		T	39 (16.8)	56 (36.1)	

*VEGFA*, vascular endothelial growth factor A; HWE, Hardy-Weinberg equilibrium.

a The p-value is for chi-square test.

**Table 4.** The OR of *VEGFA* gene polymorphisms in patients with preterm birth and control group

Maker	Preterm birth vs. control		
	OR	95% CI	p-value^a^
*VEGFA* rs699947			
Genotype			
CC	1.00	-	Reference
CA	0.70	0.423–1.165	0.170
AA	0.97	0.353–2.645	1.000
Allele			
C/A	0.92	0.622–1.350	0.407
Dominant (CC/CA + AA)	0.74	0.456–1.203	0.225
Recessive (CC + CA/AA)	0.83	0.311–2.227	0.714
Overdominant (CC + AA/CA)	0.66	0.409–1.076	0.157
rs2010963			
Genotype			
CC	1.00	-	Reference
CG	0.81	0.430–1.525	0.513
GG	1.40	0.705–2.787	0.335
Allele			
C/G	0.82	0.578–1.148	0.241
Dominant (CC/CG + GG)	1.00	0.550–1.807	1.000
Recessive (CC + CG/GG)	1.63	0.961–2.751	0.069
Overdominant (CC + GG/CG)	0.66	0.409–1.076	0.096
rs3025039			
Genotype			
CC	1.00	-	Reference
CT	0.78	0.457–1.333	0.364
TT	1.68	0.365–7.716	0.501
Allele			
C/T	0.92	0.585–0.437	0.704
Dominant (CC/CT + TT)	0.83	0.496–1.395	0.484
Recessive (CC + CT/TT)	0.76	0.450–1.303	0.325
Overdominant (CC + TT/CT)	1.81	0.397–8.246	0.437

*VEGFA*, vascular endothelial growth factor A; HWE, Hardy-Weinberg equilibrium.

a The p-value is for chi-square test.

**Table 5.** Combined three genotypes of *VEGFA* polymorphisms in the patients with preterm birth and control group

Marker	Combination	No. (%)		p-value^a^	OR (95% CI)
		Preterm birth (n = 116)	Control (n = 155)		
*VEGFA* rs699947	CC - CC - CC	14 (12.1)	22 (14.2)	-	Reference
rs2010963	CC - CC - CT	7 (6.0)	7 (4.5)	0.475	1.57 (0.453–5.450)
rs3025039	CC - CC - TT	2 (1.7)	1 (0.6)	0.347	3.14 (0.260–37.992)
	CA - CC - CC	1 (0.9)	1 (0.6)	0.754	1.57 (0.091–27.212)
	CA - CC -CT	0	1 (0.6)	0.316	NA
	CA - CC - TT	0	0	1.000	NA
	AA - CC - CC	0	0	1.000	NA
	AA - CC - CT	0	0	1.000	NA
	AA - CC - TT	0	0	1.000	NA
	CC - CG - CC	23 (19.8)	32 (20.6)	0.781	0.89 (0.375–2.088)
	CC - CG - CT	6 (5.2)	9 (5.8)	1.000	0.96 (0.279–3.27)
	CC - CG - TT	0	0	1.000	NA
	CA - CG - CC	13 (11.2)	27 (17.4)	0.561	1.32 (0.515–3.389)
	CA - CG -CT	6 (5.2)	14 (9.0)	0.506	0.67 (0.209–2.165)
	CA - CG - TT	2 (1.7)	2 (1.3)	0.67	1.57 (0.198–12.470)
	AA - CG - CC	1 (0.9)	0	0.147	NA
	AA - CG - CT	0	0	1.000	NA
	AA - CG - TT	0	0	1.000	NA
	CC - GG - CC	12 (10.3)	5 (3.2)	0.031^*^	3.77 (1.091–13.032)
	CC - GG - CT	3 (2.6)	2 (1.3)	0.369	2.36 (0.349–15.927)
	CC - GG - TT	0	0	1.000	NA
	CA - GG - CC	13 (11.2)	13 (8.4)	0.384	1.57 (0.567–4.357)
	CA - GG -CT	6 (5.2)	10 (6.5)	1.000	0.94 (0.280–3.174)
	CA - GG - TT	0	0	1.000	NA
	AA - GG - CC	4 (3.4)	2 (1.3)	0.203	3.14 (0.507–19.492)
	AA - GG - CT	3 (2.6)	7 (4.5)	0.606	0.67 (0.149–3.047)
	AA - GG - TT	0	0	1.000	NA

*VEGFA*, vascular endothelial growth factor A; OR, odds ratio; CI, confidence interval.

* p < 0.05.

a The p-value is for chi-square test.

**Table 6.** Minor allele frequencies of *VEGFA* polymorphisms in worldwide populations

	rs699947	rs2010963	rs3025039
Korean	A = 0.2631	C = 0.4266	T = 0.1883
Europe	A = 0.4882	C = 0.3082	T = 0.1213
East Asian	A = 0.2817	C = 0.4077	T = 0.1696
South Asian	A = 0.3970	C = 0.2490	T = 0.1090

Minor allele frequencies based on NCBI dbSNP. *VEGFA*, vascular endothelial growth factor A.

## Supplementary Materials

Supplementary Table 1. Haplotype frequencies of VEGFA polymorphisms in the patients with preterm birth and control group
