# Supplementary material for: Lack of association between the VEGFA gene polymorphisms and preterm birth in Korean women
Source: Genomics Inform. 2023 Sep 27;21(3):e29. doi: 10.5808/gi.22064 (PMC10584649; doi:10.5808/gi.22064)
Supplement: Supplementary Table 1. — Haplotype frequencies of VEGFA polymorphisms in the patients with preterm birth and control group [file gi-22064-Supplementary-Table-1.pdf]

**Supplementary Table 1.** Haplotype frequencies of *VEGFA* polymorphisms in the patients with preterm birth and control group

| Marker | No. (%) | | p-value[a] | Adjusted p-value[b] | OR (95% CI) |
|---|---|---|---|---|---|
| | Preterm birth (n = 116) | Control (n = 155) | | | |
| rs2010963 rs699947 | | | | | |
| C-C | 48 (41.4) | 73 (47.1) | - | - | Reference |
| C-A | 2 (1.7) | 1 (0.6) | 0.346 | 1.000 | 3.042 (0.029 - 34.479) |
| G-C | 39 (33.6) | 39 (25.2) | 0.152 | 0.455 | 1.521 (0.857 - 2.700) |
| G-A | 27 (23.3) | 42 (27.1) | 1.000 | 1.000 | 0.978 (0.534 - 1.791) |

*VEGFA*, vascular endothelial growth factor A; OR, odds ratio; CI, confidence interval.

[a]The p-value is for chi-square test.

[b]The p-value is adjusted by Bonferroni correction.
